# The Breast Cancer Stem Cells Traits and Drug Resistance

**DOI:** 10.3389/fphar.2020.599965

**Published:** 2021-01-28

**Authors:** Qinghui Zheng, Mengdi Zhang, Fangfang Zhou, Long Zhang, Xuli Meng

**Affiliations:** ^1^ Department of Breast Surgery, Zhejiang Provincial People’s Hospital, Hangzhou, China; ^2^ MOE Laboratory of Biosystems Homeostasis and Protection and Innovation Center for Cell Signaling Network, Life Sciences Institute, Zhejiang University, Hangzhou, China; ^3^ Institutes of Biology and Medical Science, Soochow University, Suzhou, China

**Keywords:** breast cancer stem cells, drug resistance, clinical therapy, surface markers, breast cancer stem cell signaling pathways

## Abstract

Drug resistance is a major challenge in breast cancer (BC) treatment at present. Accumulating studies indicate that breast cancer stem cells (BCSCs) are responsible for the BC drugs resistance, causing relapse and metastasis in BC patients. Thus, BCSCs elimination could reverse drug resistance and improve drug efficacy to benefit BC patients. Consequently, mastering the knowledge on the proliferation, resistance mechanisms, and separation of BCSCs in BC therapy is extremely helpful for BCSCs-targeted therapeutic strategies. Herein, we summarize the principal BCSCs surface markers and signaling pathways, and list the BCSCs-related drug resistance mechanisms in chemotherapy (CT), endocrine therapy (ET), and targeted therapy (TT), and display therapeutic strategies for targeting BCSCs to reverse drug resistance in BC. Even more importantly, more attention should be paid to studies on BCSC-targeted strategies to overcome the drug resistant dilemma of clinical therapies in the future.

## Introduction

Breast cancer (BC) is one of the most common cancers diagnosed among women and ranked as the second cause of cancer-related death among women, after lung cancer (DeSantis et al., 2019; Siegel et al., 2019). There are various types of BC therapeutic strategies, such as breast surgery, radiotherapy (RT), chemotherapy (CT), endocrine therapy (ET), targeted therapy (TT), and others, which are based on the types of tumor pathologies. For example, breast-conserving/mastectomy surgery and adjuvant CT are applied to treat early BCs. Antitumor drugs are utilized alone or in combination to reduce the risk of BC recurrence. For ERα-positive and Her2-positive tumors patients, hormone therapy and targeted therapy, respectively, conduce to significant prognosis improvements. Additionally, chemotherapy is considered the best option in advanced triple-negative BC (TNBC). These treatment options have contributed to a BC death rate decline over the past three decades (DeSantis et al., 2019). Hence, therapies improvement is a milestone in BC therapy.

**FIGURE 1:**
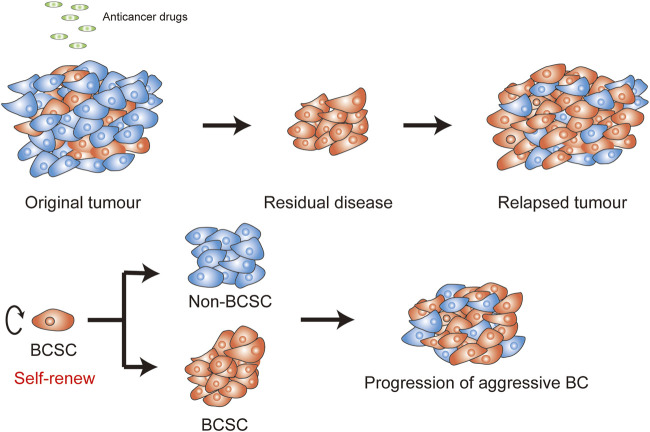
Self-renew in BCSCs. **(A)** Anticancer drugs are often utilized for treat BC, efficiently targeting breast cancer cells (BCCs) (blue cells), but not BCSCs (orange cells). The residual stem-like cell populations can drive a more aggressive BC and trigger recurrence. **(B)** BCSCs can form a new tissue by the balance of renewal and divisions.

However, many BC patients still experience poor drug response and tumor recurrence in clinical observation (Harbeck and Gnant, 2017). Some BC cells exhibit intrinsic drug-resistance, while others are initially drug-sensitive, but acquire resistance to anticancer drugs (Abad et al., 2020). These drug failures are considered as chemoresistance in BC cells, owing to the survival of a special population of heterogeneity cells in tumors which possess drug-resistance features (Eiro et al., 2019). These heterogeneity cells are known as residual disease and can eventually lead to recurrence (Figure 1).

Cancer stem cells (CSCs), which were discovered and developed over the past decades, play a major role in drug-resistance and relapse of solid tumors (Reya et al., 2001; Clarke et al., 2006). Besides drug-resistance, previous studies have showed that cancer initiation (Barker et al., 2009), progression (Lytle et al., 2018), and metastasis (Oskarsson et al., 2014) could also be induced by CSCs (Nalla et al., 2019). CSCs play a similar role in BC. Al-Hajj first isolated BC stem cells (BCSCs) with specific markers (EpCAM+/CD44^+^/CD24^-^) which have the potential to lead to bulk tumors *in vivo* (Al-Hajj et al., 2003). Targeting BCSCs, in any hypotype of BC: luminalA, luminalB, human epithelial growth factor receptor 2 (HER2) overexpression, or TNBC, is the key therapy approach to reverse drug resistance (Dey et al., 2019). Therefore, we need to understand the role of BCSCs in drug-resistance mechanisms, which will overcome the drug-resistance problem and promote BC prognosis.

Here, first we summarize the BCSC markers and signaling pathways that are possible therapeutic targets for drug resistance. More importantly, we focus on the mechanism of resistance to specific drugs, such as anthracycline, taxane, tamoxifen, trastuzumab, among others. Lastly, novel studies about emerging therapies of reversing drug resistance by targeting BCSCs are discussed. We insist that the important breakthroughs in the field of BCSCs research will help researchers effectively find and target BC resistance mechanism and, ultimately, help patients achieve a favorable prognosis.

## Central Surface Markers in Breast Cancer Stem Cells

**FIGURE 2:**
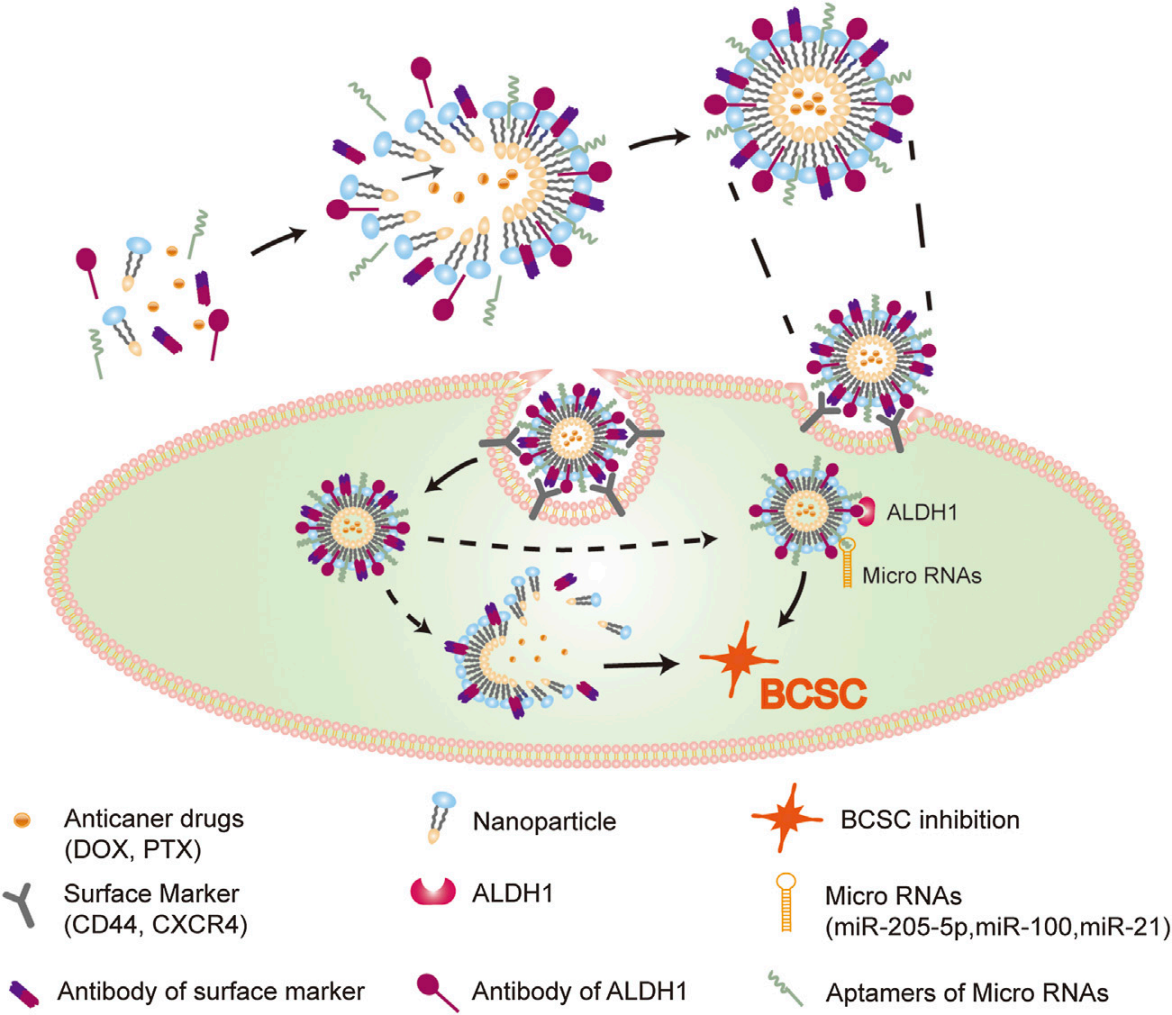
Effective drugs delivery system in BCSCs. Nanoparticles, which modified by surface markers (CD44, CXCR4) ligands and intracellular molecular (micro RNAs, ALDH1) ligands, loaded with anti-cancer drugs, efficiently targeting BCSCs. The delivery system shows effectively reversal of drug resistance through dual inhibition of BCSCs via repressing both ex- and intracellular tumorigenic markers.

BCSCs surface biomarkers are utilized for identifying or isolating BC. However, emerging studies show that different surface markers determine different BCSCs (Dey et al., 2019; Sridharan et al., 2019); the functions of BCSCs are based on the type of markers they contain. The key surface markers of BCSCs and their functions in BC are listed in Table 1. Novel drugs are being designed to target these markers for regulating the activation of BCSCs in order to achieve an efficient response to anti-BC treatment (Figure 2). Thus, we list the central surface markers in BCSCs and their known functions in BCSCs regulation.

**TABLE 1:** The BCSCs Surface markers in significant literatures.

Surface markers	Mediated signaling	Function in BC
CD44 (Al-Hajj et al., 2003)	PI3K/AKT signaling (Ghatak et al., 2002)	Forming tumors (Al-Hajj et al., 2003), promoting metastasis (Hill et al., 2006), associated with (BRCA1) mutational status (Bane et al., 2013) Predicting prognosis (Bane et al., 2013), stimulating migration (Bourguignon et al., 2003), promoting cell adhesion (Ponta et al., 2003), promoting. Cell growth, survival, and invasion (Louderbough and Schroeder, 2011)
	NF-κB signaling (Cho et al., 2015)	
	CREB/TGF-β2 signaling (Ouhtit et al., 2018)	
ALDH1 (Ginestier et al., 2007)		Associated to tumor-initiating characteristics (Ginestier et al., 2007), promoting self-renewal (Ginestier et al., 2009), As target for BCSCs-targeted therapy (Angeloni et al., 2015) Predicting prognosis 11823860 (Alexe et al., 2006; van’t Veer et al., 2002) promoting metastasis (Marcato et al., 2011)
CD133	IL6/Notch3 signaling (Sansone et al., 2016)	Regulation of ET-resistant (Sansone et al., 2016) promoting self-renewal (Sansone et al., 2016) BCSCs identification (Bai et al., 2018)
		CD133 aptamers or CD133-targeted drug delivery system for BCSCs-targeted therapy (Shigdar et al., 2013; Swaminathan et al., 2013)
EpCAM		Regulation of migration and metastasis (Baccelli et al., 2013) promoting chemoresistance (Wang T. et al., 2015)
ABCG2		Promoting BC chemoresistance, tumorigenicity and metastasis (Bai et al., 2018), Sorting BCSCs from BRCA1-mutated BC cells (Leccia et al., 2014)
GD2		Aassociated with GD3S-mediated EMT (Liang et al., 2017), promoting tumorigenicity and metastasis (Battula et al., 2017),BCSCs. identification (Bai et al., 2018)
CXCR4	SDF-1/CXCR4 signaling (Yi et al., 2014)	Promoting metastasis (Muller et al., 2001), promoting migration or invasion (Luker et al., 2012)

**TABLE 2:** Resistance mechanisms for major drugs in BC therapy.

Drug resistance	Related markers or pathways	Mode of action	*In vitro* or *in vivo* or clinical trial	References
Resistance to chemotherapy				
Paclitaxel	JAK/STAT3-CPT1B-FAO-LPEs	Paclitaxel resistance is regulated by JAK/STAT3-CPT1B-related fatty acid oxidation in BCSCs	*In vitro*	Wang T. et al. (2018)
	MYC/MCL1-(mtOXPHOS) -(ROS) -HIF-1α	paclitaxel resistance is regulated by mitochondrial oxidative phosphorylation (mtOXPHOS) via MYC/MCL1-(mtOXPHOS) - (ROS)-HIF-1αpathway in BCSCs	*In vitro*	(Lee et al., 2017)
	ROS-HIF1/2α-IL-6/IL-8/MDR1	Chemotherapy-induced HIF activity enriched the BCSCs through IL-6 and IL-8 signaling and increased the expression of multidrug resistant proteins (MDR1)	*In vitro*	(Samanta et al., 2014)
	EIF2AK3/EIF2AK4-pEIF2S1-ATF4	Paclitaxel resistance is regulated by redox homoeostasis (ISR) in BCSCs	*In vitro* and *in vivo*	Chen et al. (2019a)
	Jagged2- microRNA-200	Jagged2 promotes the maintenance of BCSCs properties and paclitaxel resistance by regulating the over-expression of microRNA-200	*In vitro* and *in vivo*	Li C. Y. et al. (2018)
	IGF2BP3/CD44-IGF2- Hedgehog signalling	CD44-expressing fibroblasts can inhibit paclitaxel-induced apoptosis, leading to paclitaxel resistance	*In vitro*	Liu Y. et al. (2017)
	ABCB1	Amplification of chromosome region 7q21 coordinated the overexpression of resistance-related proteins and caused cancer cells to develop multidrug resistance.	—	(Genovese et al., 2017)
	ABCB1/ABCG2	Atp binding cassette (ABC) transporter linked to paclitaxel resistance	—	Arnason and Harkness (2015); Robey et al. (2018)
	MTDH/NF-κb signalling	MTDH reduces NF-κB expression and increases p65/p-p65 expression, causing paclitaxel resistance	*In vitro* and *in vivo*	(Yang et al., 2018)
	ERα-activated-DNMT1/DNMT3b	DNMT1 induces DNA methylation and promotes paclitaxel resistance	*In vitro*	(Si et al., 2016)
	MENA/MAPK signalling	MENA subtype expression changes microtubule status after paclitaxel	*In vitro* and *in vivo*	(Oudin et al., 2017)
Anthracyclines	SLC34A2-Bmi1-ABCC5 signalling.	Increases the expression of SLC34A2 in BCSCs induces chemotherapy resistance to Dox through the slc34a2-bmi1-abcc5 signaling pathway.	*In vitro* and *in vivo*	(Ge et al., 2016)
	Glucosylceramide synthase (GCS)	The overexpression of GCS in BC cells is induced by Dox and is related to the pluripotency of BCSCs	*In vitro* and *in vivo*	(Bhinge et al., 2012)
	HIF-2α/BCRP axis	Chemotherapy-mediated HIF-2α directly promotes the expression of BCRP and coordinates the ability of anti-dox in BCSCs.	*In vitro*	(He et al., 2019)
	TOPOII	Mesenchymal stem cells can effectively repair DNA double-strand breaks induced by topoisomerase inhibitors	*In vitro*	(Nicolay et al., 2016)
	ANXA3/NF-κb signalling pathway	ANXA3 overexpression increased the heterogeneity and adriamyclins resistance in BCSCs by the actvation of NF-κB signalling pathway.	*In vitro* and *in vivo*	(Du et al., 2018)
	KLF4 signalling pathway	Adriamyclins chemotherapy increased the expression of CD133, ALDH1A1, ABCG2, and the maintenance of BCSCs characteristics	*In vitro* and *in vivo*	(Li et al., 2017)
Resistance to endocrine Therapy				
Tamoxifen	CD44 + CD24-	High CD44 + /CD24 - ratio is displayed in tamoxifen resistant BC	*In vitro*	(Wang et al., 2012)
	Stem cell markers	Upregulates ALDH, Sox2,Oct4, and CXCR4 in tamoxifen resistant cells	—	(Piva et al., 2014; Gwak et al., 2017; Raffo et al., 2013; Dubrovska et al., 2012; Wang et al., 2012)
	ER signaling pathway	Mutations in the ERα promote the generation of BCSCs markers and induce tamoxifen resistance	*In vitro*	(Gelsomino et al., 2018)
	PI3K/AKT/mTOR signalling	Promotes self-renewal and survival of BCSCs in tamoxifen resistant cells	*In vitro*	(Gargini et al., 2015; Kolev et al., 2015)
	IGFR	Maintains BCSCs surface markers expression and tumorigenesis by the activation of AKT	*In vitro* and *in vivo*	(Chang et al., 2013)
	Wnt/β-catenin pathway	Activation along with the enrichment BCSCs in tamoxifen resistant	*In vitro*	(Loh et al., 2013; Angeloni et al., 2015)
	Notch signalling	Develops tamoxifen resistance via regulating BCSCs	*In vitro*	(Magnifico et al., 2009; Yun et al., 2013)
	IL6/STAT3	Promotes BCSCs and stimulates tamoxifen resistance	*In vitro*	(Wang et al., 2012)
	Hh pathway	Maintains the self-renewal of BCSCs in response to tamoxifen treatment	*In vitro* and *in vivo*	(Ramaswamy et al., 2012)
	TGF-β	Generates the phenotype of BCSCs and induces tamoxifen resistance	*In vitro*	(Liu et al., 2012; Kopp et al., 1995)
Fulvestrant	ER signaling pathway	ERβ as a therapeutic target to in BCSCs to re-sensitizes fulvestrant and tamoxifen resistant cells	*In vitro* and *in vivo*	(Ma et al., 2017)
	Stem cell markers	Up-regulation of ALDH1, NANOG, OCT4 and SOX2 in response to tamoxifen or fulvestrant	*In vitro*	(Lillo et al., 2017)
	NOTCH	Maintains the activity of BCSCs to resistant fulvestrant	*In vitro* and *in vivo*	(Simoes et al., 2015)
AI	CD44/CD24	High CD44 + /CD24 - ratio is demonstrated in AI-resistant cell	*In vitro*	(Wang et al., 2013; Uchiumi et al., 2019)
Letrozole	PI3K/Akt/mTOR signalling pathway	BCBSs-mediated letrozole resistance by regulating PI3K/Akt/mTOR signaling pathway	*In vitro*	Liu Y. et al. (2019)
		Promotes BCSCs enrichment in MCF-7, and inversing by mTOR inhibitors	*In vitro* and *in vivo*	(Liu et al., 2014)
	JNK signaling pathway	Promotes the stemness of BC cells to cause aromatase inhibitors resistance	*In vitro*	(Pelekanou et al., 2018)
	Stem cell markers	Up-regulation of ALDH1, Oct 4, SOX2, and nanog in resistance cells	*In vitro*	(Nasr et al., 2018)
	HER2 signaling	Mediates AI resistance via regulation of stem cell markers, such as breast cancer resistance protein (BCRP)	*In vitro*	(Gilani et al., 2012)
Letrozole or exemestane	HIF-1α	Improves the generation of BCSCs to resistant to letrozole and exemestane	*In vitro*	(Kazi et al., 2014)
exemestane	RTKs pathway	Accumulates stemn-like cancer cells and resistant to exemestane	—	(Farahmand et al., 2018)
palbociclib	PI3K/Akt/mTOR signalling	Increases the ability of stemness and migration in palbociclib-resistant BCSCs	*In vitro*	Chen et al. (2019b)
	IL-6/STAT3 pathway	Promotes BCSCs enrichment	*In vitro* and *in vivo*	(Kettner et al., 2019)
	EMT	Promotes the capacity of migration and invasion via regulating BCSCs in CDK4/6 inhibitor-resistant BC	*In vitro* and *in vivo*	(Kettner et al., 2019; Pandey et al., 2019)
Resistance to Targeted Therapy				
Trastuzumab	PI3K/AKT signalling	Induces trastuzumab resistance via activating PI3K/AKT pathway in BCSCs	*In vitro* and *in vivo*	(Choi et al., 2019)
	JAK/STAT3 signalling	STAT3 activation increases CSCs properties then results in trastuzumab resistance	*In vitro*	(Chung et al., 2014)
	Wnt/β-catenin signalling	Over-activating wnt signalling pathway promotes CSCs then leads to trastuzumab resistance	*In vitro*	(Wu et al., 2012; Choi et al., 2019)
	MUC1	The number of MUC1 increases in trastuzumb resistant cell lines while anti-MUC1 inhibits CSCs proliferation	*In vitro*	(Sand et al., 2020)
	CD44^+^/CD24^-^	Acts as a predictor of poor response to trastuzumab	Clinical trial	(Seo et al., 2016)
Trastuzumab				
Lapatinib	TGFβ- Smad	Enhances the CSCs traits then leads to resistance of targeted therapy	*In vitro*	(Chihara et al., 2017)
Lapatinib	PI3K/AKT signalling	Directly represses HER2 and indirectly inhibits EGFR	*In vitro*	(Iorio et al., 2009; De Cola et al., 2015)
	CD44^+^/CD24^-^	Decreases the sensitivity of HER2+ BC cells to lapatinib	*In vitro*	(Hosonaga et al., 2014)

MTDH, Metadherin; ISR, The integrated stress response; MUC1, Mucin 1.

### CD44

CD44 was initially used to isolate BCSCs from tumors. Meanwhile, BC cells with an overexpression of CD44 marker, known as BC-initiating cells (BCIC), showed tumorigenic ability *in vivo* (Ponti et al., 2005). CD44 is a cell membrane receptor for hyaluronan acid (HA) (Bourguignon et al., 2004). HA-CD44 interaction play an important role in inhibiting metastasis (Lv et al., 2018a; Bourguignon, 2019), reversing drug resistance (Liu J. et al., 2019), and suppressing invasion (Sarkar et al., 2019) among BC cells. For instance, The binding of CD44 and HA activated c-Src-Twist/miR-10b/RhoGTPase-ROK signaling, that are associated with the activation of the PI3K/AKT-dependent invasion and metastasis in cancers (Bourguignon et al., 2010). Furthermore, the high expression of CD44 is essential for BC multidrug resistance by regulation of the chemoresistance receptor through stimulation of signal transducer and activator of transcription 3 (STAT3) pathway (Louderbough and Schroeder, 2011). Moreover, the interaction of the cleaved product of CD44 (CD44ICD) and cAMP-response element binding protein (CREB) can up-regulate fructose-2,6-bisphosphatase 4 (PFKFB4) expression, which activates glycolysis and impoves BC stemness (Gao et al., 2018). CSCs are associated with tumor metastasis and invasion.

Conversely, CD44 is also utilized as a targeting marker of HA-drug-nanocomposite complex. The combination of HA and docetaxel (DTX), loaded in polymeric nanoparticles (NPs), improved the effect of drug delivery by targeting CD44+^high^ BC cells (Gaio et al., 2020). Similarly, a HA-NPs complex loaded with paclitaxel (PTX) was well designed to target CD44 for improvement of chemotherapeutic effects in metastatic cancer (Lv et al., 2018b). These results demonstrate the important role of CD44 in BC stemness, invasion, metastasis, and drug resistance. We should aim at significantly reversing drug resistance through the use of nano-drug combinations, improving drug efficacy, and ultimately, ensuring a favorable prognosis.

### CD133

CD133, known as Prominin-1, is independently expressed on the surface of stem cells and various tissue tumor stem cells. Similar to CD44, CD133 BC cells show stem-like properties and are found to be enrich in basal-like, triple negative, HER2+ or luminal tumors (Borgna et al., 2012).

xenograft-initiating CD44^pos^CD49f^high^CD133/2^high^ cells among ER-negative tumors were capable of forming ER-negative tumors (Meyer et al., 2010), supporting the evidence that CD133 is an identifier molecule for BCSCs with high aggressive properties.

The accumulation of CD133^high^ BCSCs aggravated BC and tended to induce drug-resistance (Bousquet et al., 2017), proliferation (Brugnoli et al., 2017), vasculogenic mimicry (Liu et al., 2013), invasion, and metastasis (Bock et al., 2014). For instance, heterogeneous BC cells with CD133 marker displayed resistance to drugs and the potential to form a mass in NOD/SCID mice (Wright et al., 2008). Moreover, CD133^high^ BCSCs were enriched in the tumors of hormonal therapy (HT)-resistant BC, forming metastatic luminal BC by self-renewal during HT (Sansone et al., 2016). The capability of self-renewal can be switched through re-expression of estrogen receptor (ER) by inhibition of IL6R/IL6-Notch pathways (Sansone et al., 2016). Furthermore, a ribonucleoprotein complex (LncRNA MALAT1 and HUR) down-regulated the expression of CD133+ phenotype and inhibited the stem cell properties of BCSCs, leading to tumorigenesis and metastasis failure both in MCF-7 and MDA-MB-231 (Latorre et al., 2016), revealing the indirect mechanism of CD133 and drug resistance in BC.

Recently, a novel CD133-targeting drug delivery system that uses nanoparticles loaded with drugs was reported. An anti-CD133 antibody into nanoparticles loaded with paclitaxel, increased the accumulation of paclitaxel in CD133+ cells, decreased the population of BCSCs, and inhibited the tumorigenic ability *in vivo* (Swaminathan et al., 2013). This implies that CD133-targeting will contribute to the development of BCSC-targeting therapeutics to reverse drug resistance.

### EpCAM

EpCAM, a type I transmembrane glycoprotein, is known as a phenotype of epithelial tumors and is overexpressed in BCSCs (Munz et al., 2009). EpCAM can promoting BCSCs survival through the activation of Wnt/β-catenin signaling pathway (Sena and Chandel, 2012). It can also promote adhesion between epithelial cells, playing an important role in migration and metastasis. For example, EpCAM^+^ disseminated tumor cells (DTCs), isolated from the peripheral blood of BC patients, contained a class of metastatic initiating BC cells that could cause bone, lung, and liver metastases in NOD-SCID mice (Baccelli et al., 2013). Moreover, EpCAM still plays an important role in reversing resistance. For instance, Survivin silencing, mediated by EpCAM aptamer, can make BCSCs sensitive to doxorubicin, leading to the reversal of resistance, which indicates that this novel strategy is an effective method to reverse drug resistance in BC (Wang T. et al., 2015).

### ALDH1

ALDH1 is an NAD(P)+ dependent enzyme that mediates the oxidation of intracellular aldehydes into carboxylic acids. ALDH1 acts as a common marker of both normal and malignant breast stem cells, especially in BCSCs. ALDH1-high activity is an independent predictor of progression and poor survival of BC patients (Ginestier et al., 2007). Moreover, CD44+/CD24−/ALDH1+ MDA-MB-231 and CD44+/CD133−/ALDH1+ MDA-MB-468 BC cells demonstrated stronger tumorigenic and metastatic capabilities than ALDH1^low^CD44^low^ BC cells (Croker et al., 2009).

However, ALDH activity of BCSCs was mainly dependent on ALDH1A3, rather than on ALDH1A1 (Marcato et al., 2011), further enhancing the understanding of specific targets of BCSCs. The main explanation for this difference is that the expression level of ALDH1A1 in breast epithelial cells is lower than that of ALDH1A3. The strong association between LDH1A3 high expression and metastasis in BC patients was also reversed to confirm the importance of ALDH1A3 in BC. Contrarily, NOTCH signaling pathway increased ALDH1A1 Lys-353 deacetylation at a post-translational level through the induction of silent information regulator 2 (SIRT2) expression, promoting tumorigenesis and tumor growth in a BC model (Zhao et al., 2014). Conversely, inhibition of ALDH activity resulted in drug (doxorubicin/paclitaxel) resistance reversal in ALDH^high^ CD44^+^ BCSCs (Croker and Allan, 2012). Therefore, these studies reveal that ALDH1 not only can be utilized to distinguish BCSCs, but also as a potential therapeutic target for drug resistance reversal in BC. ALDH1 regulation might be useful in explaining drug resistance in further research.

### CXCR4

As a specific receptor of stromal cell-derived factor-1 (SDF-1), CXC chemokine receptor 4 (CXCR4) is essential for BCSCs-related metastasis. The SDF-1/CXCR4 signaling pathway mediates the role of promoting the directional metastasis of CXCR4+ BCSCs. Both antibody neutralization and CXCR4 knockdown inhibited the proliferation of orthotopically transplanted breast tumor and metastasis (Muller et al., 2001). Non-metastatic BCSCs promote the transformation of non-BCSCs to CXCR4^+^ BCSCs in BC tissues (Mukherjee et al., 2016). Besides, CXCR4+ BCSCs displayed decreased vimentin and increased E-cadherin, indicating the occurrence of epithelial-mesenchymal transitions (EMT). These findings demonstrate that CXCR4^+^ BCSC triggered EMT-related metastasis.

BC metastasis is closely related to drug resistance, so CXCR4 may be a key factor of reversins drug resistance. CXCR4 is also closely related with tumor microenvironmental changes. CXCR4 is highly expressed in BC metastases; thus, I.X. found that suppressed CXCL12/CXCR4 signaling pathway or silenced CXCR4 in BCSCs sensitizes BC to immune checkpoint blockers, inhibiting metastasis reversing drug-resistance in BC (Chen I. X. et al., 2019). In a similar mechanism, DPP-4 inhibitors were found to reverse drug-resistance via ABC transporters-mediated CXCL12/CXCR4/mTOR/TGFβ axis in BC cells (Li et al., 2020). An innovative strategy, consisting of an oncolytic virus loaded with a CXCR4 antagonist, was utilized for targeting the CXCL12/CXCR4 signaling pathway, being remarkably effective in primary and metastatic BC (Gil et al., 2013). Furthermore, the activation of SDF-1/CXCR4 signaling pathway can increase the phosphorylation of 60 proteins with migration or invasion properties in BC, which might be key mediators for CXCR4-induced BCSCs proliferation (Yi et al., 2014). These evidences emphasized CXCR4 as a therapeutic target to inhibit microenvironment-induced stemness and the appearance of metastatic phenotypes and made it possible to eradicate the activation of CXCR4-related signaling pathway, decreasing the proportion of CXCR4+ BCSCs.

### ABCG2

As a known drug-resistant protein, ABCG2 is highly expressed in BC resistant cells, especially in resistance-related BCSCs. Sun found that stem-like CD44^+^CD24^−/low^ cells isolated from several BC cell lines, such as SK-BR-3, MDA-MB-231, and MCF-7 displayed a higher expression of ABCG2 than non-stem cells (Sun et al., 2015). Furthermore, ABCG2 is considered to be a more effective surface marker for BCSCs identification than CD44^+^CD24^−^ (Leccia et al., 2014). Moreover, several pieces of evidence have highlighted ABCG2 as a therapeutic target to overcome BC multidrug resistance. For instance, downregulation of either Rab5A or Rab21 increases surface expression of ABCG2 and efflux of intracellular drugs, reversing BC drug-resistance (Yousaf and Ali, 2020). Moreover, it has also been demonstrated that drug resistance can be reversed by ABCG2 modulators at a molecular level (Hasanabady and Kalalinia, 2016; Pena-Solorzano et al., 2017). However, few small molecule modulators have shown to be effective in preclinical trials. Therefore, the role of ABCG2 inhibitors in reversing resistance by mediating BCSCs should be re-examined and more *in vivo* evidence should be presented.

### GD2

GD2, a b-series ganglioside, is another cell membrane phenotype of BCSCs. Indeed, GD2^+^ BC cells, isolated from either BC cell lines or clinical tumor tissue in BC patients, show stemness. Meanwhile, it has been revealed that GD2+ cells, human mammary epithelial cells-derived GD2+ cells, were highly CD44^+^CD24^−^ (Battula et al., 2012). GD3 synthase, a rate-limiting enzyme, regulates the synthesis of GD2 and is considered a kind of therapeutic target for BCSCs. GD3S was positively correlated with the expression of GD2+ in BCSCs, and the low expression of GD3S not only resulted in the decreased expression of GD2+, but also disrupted EMT-mediated tumor formation ability of BC cells (Liang et al., 2017). Consistently, another study indicated that the high expression of GD3S was closely associated with the activation of nuclear factor kappa-B (NF-κB) in GD2^+^ BCSCs. The Inhibition of NF-κB signal can significantly reduce the expression of GD3S and the proportion of GD2^+^ BCSCs, abolishing the capability of BCSCs to metastasize (Battula et al., 2017). Based on the correlation between BCSCs, GD3S, and GD2, the development of GD3S-related signals as a novel therapeutic target may induce BCSCs to reverse drug resistance.

## Central Signaling Pathways in Breast Cancer Stem Cells

**FIGURE 3:**
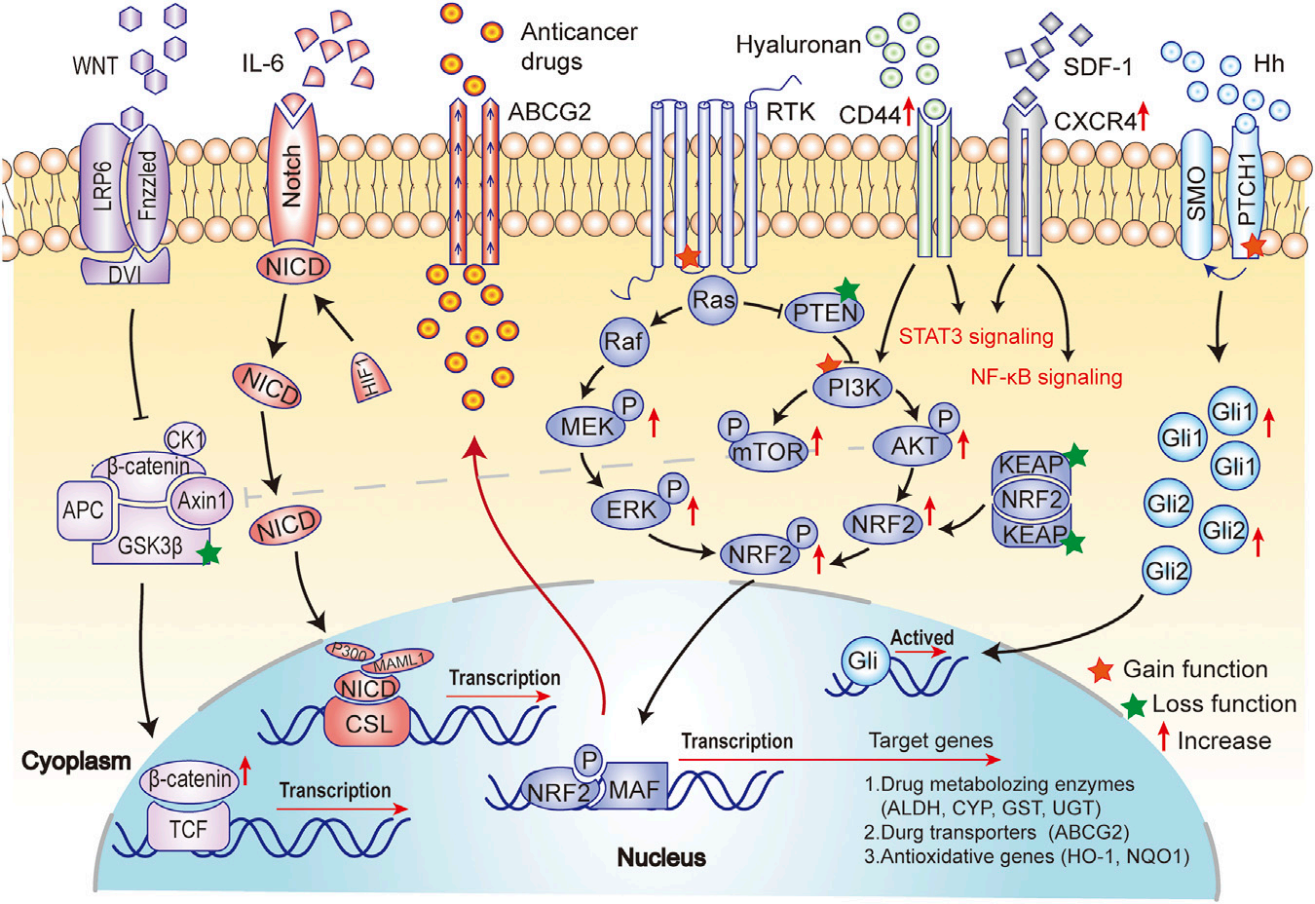
Major mechanisms of drug resistance in BCSCs. Drug resistance is not only a result of the activation of the self-renewal (Notch and Hh signaling pathway) and anti-apoptotic (PI3K/AKT/mTOR signaling) in BCSCs, but also a consequence of the promotion of metastasis (EMT and Wnt/β-catenin signaling pathway), anti-oxidative activity (NRF2 signaling) and ATP-binding cassette (ABC) transporter (ABCG2) activity in BCSCs.

As mentioned above, surface markers play an important role in maintaining the stemness of BCSCs, but they can't work independently of intercellular signaling pathways. Here, we continue to describe the activation of several key intracellular signaling pathways in BC, as a result of gene mutation, epigenetic modifications, or tumor microenvironment changes, which generate drug resistance-related BCSCs. Therefore, understanding the relevant pathways can contribute to better understand the characteristics of BCSCs and determine the research direction of reversing drug resistance targeted therapy. Major mechanisms of drug resistance in BCSCs are shown in Figure 3

### Notch Signaling Pathway

Notch signaling pathway is one of the regulative mechanisms of BCSCs’ self-renewal and survival. Cytokine IL-6 regulates Notch signaling, and the increase of IL-6 was detected in BC treated with HT, activating the Notch3 signaling in BC cells. The activation of Notch3 signaling enables BC cells to self-renew instead of the ER-dependent survival mechanism, thus impacting clinical efficacy of HT. However, inhibiting Notch signaling significantly reduced the self-renewal ability of CD133^high^ER^low^ BCSCs in HT-resistant cells (Sansone et al., 2016). Similarly, another study indicated that the combination of MK-0752 (gamma secretase inhibitors) and Tocilizumab (IL6R antagonist) remarkably decreases the proportion of BCSCs and inhibits cell proliferation or tumor growth in BC, through Notch3 signaling pathway (Wang D. et al., 2018).

Moreover, emerging evidence suggested that BCSCs mediate drug resistance in BC through Notch-related signaling pathway. For example, the activation of Notch signaling pathway promotes the appearance of stem cell phenotype in ERα/ESR1^+^ BC cell lines and causes drug resistance to ET for BC (Gelsomino et al., 2018). Consistently, the activation of JAG1-NOTCH4 signaling pathway stimulates BCSCs activity and generates anti-estrogen resistance in BC (Simoes et al., 2015). In particular, Notch1 also mediated trastuzumab resistance in BCSCs by inhibiting PTEN expression to cause the activation of ERK1/2 signaling. Notch1-PTEN-ERK1/2 signaling might be a target for the novel therapy strategies of combining anti-Notch1 and anti-MEK/ERK to reverse trastuzumab resistance (Baker et al., 2018).

### Wnt/β-Catenin Signaling Pathway

Wnt/β-catenin signaling pathway also plays an important role in BCSCs self-renewal. A previous study has shown that Wnt/β-catenin signaling pathway was deemed as a key mechanism of Sam68- mediated self-renewal in BC cells (Wang L. et al., 2015). Another study displayed that Gomisin M2 remarkably inhibited BCSCs self-renewal by suppressing the Wnt/β-catenin signaling pathway (Yang Y. et al., 2019). Compared to other cells, the higher level of Wnt/β-catenin signaling pathway contributes to the high resistance level of BCSC. CWP232228, a small-molecule of Wnt/β-Catenin inhibitor, suppressed the proliferation of BCSCs by inhibiting β-catenin-mediated transcription (Jang et al., 2015). Furthermore, this result implied that Wnt/β-catenin might indirectly regulate drug resistance by BCSCs self-renewal or proliferation, promoting Wnt/β-catenin as a therapeutic target for BCSCs therapy in the future.

### PI3K/AKT/mTOR Signaling Pathway

The activation of the phosphatidylinositol 3-kinase (PI3K)-related signaling pathway in BCSCs was reported in recent years and can be contribute to drug resistance in BC. Mounting evidence demonstrated that PI3K/AKT/mTOR signaling pathway has an important role on ET-resistance in ER+ BC (Droog et al., 2013; Ojo et al., 2015). PI3K/Akt/mTOR signaling pathway induces BCSCs metastasis by CD44 regulation. Moreover, the interaction of PI3k and Wnt/β-catenin signaling maintain the self-renewal and stemness abilities of BCSCs (Solzak et al., 2017).

Impressively, inhibition of PI3K/AKT/mTOR signaling pathway reduces BCSC survival and self-renewal. In other words, the inhibitors of PI3K/AKT/mTOR signaling pathway impact the activity of BCSCs. For instance, IGF-1R, a stemness marker, is associated with BC disease progression. Inhibitors of IGF-1R and its downstream PI3K/Akt/mTOR signaling pathway reduced the population of BCSCs. Therefore, IGF-1R/PI3K/Akt/mTOR signaling pathways are favorable targets for the treatment of BCSCs (Chang et al., 2013). Indeed, rapalogs, such as NVP-BEZ235 and NVPBGT226, were utilized as inhibitors of both PI3K and mTOR. Meanwhile, the inhibition of PI3K by rapalogs could stimulate GLP1-mediated stem-like features in BC cell lines, as the BCSCs generated imply rapalogs resistance in BC (Posada et al., 2017). Hence, future research is necessary to elucidate the relationship between the distinct mechanisms of drug resistance and the regulation of PI3k-mediated BCSCs.

### Hedgehog Signaling Pathway

The activation of Hh signaling pathway is essential to balance tissue homeostasis and self-renewal in BC. Generally, Hh signaling pathway is up-regulated in BCSCs, which may be involved in stemness maintenance. However, there are few pieces of evidence to understand the abnormal activation of Hh signaling pathway in BCSCs. Interestingly, SHH (Sonic Hedgehog), secreted by BCSCs, regulated cancer-associated fibroblasts (CAFs) via the activation of Hh signaling (Valenti et al., 2017), promoting BCSCs proliferation and self-renewal. Furthermore, Shh (Sonic Hedgehog)-mediated Hh signaling activation gives rise to salinomycin resistance (He et al., 2015). Conversely, inhibition of the Hh signaling pathway could sensitize BCSCs to paclitaxel by cyclopamine (He et al., 2015). So far, rare inhibitors of Hh signaling pathway were applied to regulate BCSCs. Thus, further studies on the activation mechanisms of Hh signaling pathway-related stemness maintenance or resistance in BCSCs are needed to identify drugs that target Hh signaling pathway for reversing drug resistance.

## Resistance to Cancer Therapy

### Resistance to Chemotherapy

Chemotherapy is an important part of BC routine treatment. Effective neoadjuvant chemotherapy helps patients to reduce tumor burden and clinical stage and provides opportunities for breast conserving surgery. Moreover, accumulating evidence indicated that advance BC patients benefit from chemotherapy. However, recent studies showed that the phenomenon of BCSCs enrichment occurs after chemotherapy in BC. Therefore, we focus on various chemotherapeutic drugs, listing the specific relationship between BCSCs and drugs, understanding the mechanism of chemotherapy resistance and summarizing the potential therapeutic strategies to reverse drug resistance.

#### Paclitaxel Resistance

Paclitaxel, a microtubule stabilizer, is widely used in BC clinical chemotherapy. It can keep the cells in the G2/M phase to inhibit the cell cycle (Horwitz et al., 1986). Unfortunately, paclitaxel resistance is becoming a clinical challenge in BC treatment. The mechanisms of paclitaxel resistance are the following. First, paclitaxel-resistant cells have the mutant microtubule binding sites, which can impact tubulin expression. Mutations in microtubule-related proteins (e.g., βI-tubulin (Giannakakou et al., 1997) and βIII-tubulin (Magnani et al., 2006)) contributed to paclitaxel resistance. Secondly, the expression of transporters, such as ATP-binding cassette transporter MDR-1/P-gp (ABCB1) (Genovese et al., 2017), BCRP (ABCG2) (Arnason and Harkness, 2015; Robey et al., 2018), which are required for paclitaxel resistance, is abnormal. Paclitaxel-resistant cells exhibit stem-like properties (Bumbaca and Li, 2018). Some scholars insist that tumor stem cells can resist to chemotherapy, and that a higher expression of CD44+/CD24- tumors displayed greater resistance to neoadjuvant chemotherapy (Creighton et al., 2009; Marotta et al., 2011). Recently, Tanei found that ALDH1 is enriched in chemotherapy resistance cells (Tanei et al., 2009). Interestingly, ALDH1 and CD44 were utilized as important surface markers to isolate BCSCs.

Recently, with the going research between BCSCs and paclitaxel resistance, scientists often focus on the biological metabolism of BCSCs with a unique perspective. Lee discovered that the interaction of MYC and MCL1 regulated the production of reactive oxygen species (ROS) and participated in mitochondrial oxidative phosphorylation (OXPHOS), further activating the HIF-dependent hypoxia pathway and enhancing the enrichment of BCSCs and paclitaxel resistance (Lee et al., 2017). Similarly, the laboratory of Dr. Samanta investigated and verified that, after paclitaxel or gemcitabine chemotherapy, BCSCs increased activity and expression of HIF-1α and HIF-2α through the paclitaxel-ROS-HIF-IL-6/IL-8 axis after chemotherapy (paclitaxel or gemcitabine) (Samanta et al., 2014). Consequently, HIF-mediated downstream signaling pathways will become a crucial target for paclitaxel resistance in BCSCs. Ultimately, IL-6 and IL-8, paclitaxel-induced, increased BCSCs enrichment and drug resistance through the STAT3 (Marotta et al., 2011) and TGF-β pathways (Bhola et al., 2013), respectively. Thus, the intrinsic relationship between STAT3 signaling and TGF-β pathway can also be an important target to regulate BCSCs to reverse drug resistance. In summary, paclitaxel resistance is not only related to its unique metabolic pathway, but also to the biological behavior of BCSCs.

#### Anthracyclines Resistance

Anthracyclines, inhibitors of topoisomerase II (TOPO II), are a broad-spectrum chemotherapy drugs, including doxorubicin and epirubicin, which are widely used in BC chemotherapy. Nevertheless, the emergence of drug resistance often caused the failure of anthracyclines chemotherapy. Emerging studies have shown that anthracyclines could exhibit different drug resistance patterns in different parts of cells (Capeloa et al., 2020): on the cell envelope, ATP-binding cassette transporter can decrease the concentration of intracellular anthracyclines (Gottesman et al., 2002; Sun et al., 2015). In the cytoplasm, alterations in apoptosis (Gyorffy et al., 2005) and autophagy (Liu et al., 2011; Sun et al., 2011) pathways impact the cytotoxic effects of anthracyclines in the cytoplasm; in the nucleus, gene mutations regulate the expression and activation of TOPO II, inhibiting the effect of anthracyclines-induced DNA damage and promoting anthracyclines resistance (Press et al., 2011; Wijdeven et al., 2015). These resistance-related proteins or pathways above are affected by metabolism. Thus, anthracyclines metabolism impacts the sensitivity of BC to anthracyclines. Many CD44^+^ or CD133+ BCSCs are enriched in tumors under anthracyclines therapy in BC (Jia et al., 2016). Other studies have shown that BCSCs could effectively remove DNA damage caused by chemotherapeutic drugs (Nicolay et al., 2016), and that the dysregulation of Annexin A3 (ANXA3) changed the sensitivity of BCSCs to doxorubicin (Du et al., 2018). These evidences support the role of BCSCs in anthracyclines resistance, and further studies on the therapeutic targets of BCSCs to reverse anthracyclines resistance should be performed.

#### Platinum Resistance

Platinum is one of the most common drugs for advanced BC because of its DNA-damaging properties. It interacts with DNA at guanine and adenine nucleotides to form Pt−DNA nonfunctional adducts that destroy double-stranded the DNA template and inhibit the division of tumor cells. However, platinum is not considered an option if progression of disease occurs during platinum‐based chemotherapy. Unfortunately, only 47% of advanced BC patients are sensitive to platinum drugs (Sledge et al., 1988). Fortunately, mounting studies show that platinum resistance is associated with BCSCs. For instance, Disulfiram could improve the cytotoxic effect of cisplatin by reversing BCSCs-mediated cisplatin resistance. Meanwhile, Disulfiram exhibited difference ability to eliminate ROS between BCSCs and non-BCSCs (Yang Z. et al., 2019). Coincidentally, more than one researcher suggested that the stem-like BC cells are modulated by ROS (Nguyen et al., 2020; Nourbakhsh et al., 2020). These results implied that ROS could affect platinum resistance by regulating BCSCs. Besides, Xu proposed that IL‐6 enhances resistance to cisplatin via the activation of STAT3 pathway in BC (Xu et al., 2018). Although STAT3 has been shown to induce BCSCs, it is unclear that IL6/STAT3 signaling pathway may affect the resistance to platinum by BCSCs modulation.

#### Capecitabine Resistance

Capecitabine is commonly used as a chemotherapy drug for advanced second-line BC. The cytotoxic effect of capecitabine is triggered by 5'-furan and thymidine phosphorylase. Therefore, low activity of thymidine phosphorylase led to capecitabine resistance in tumor tissues (Ishikawa et al., 1998). However, few pieces of evidence indicated the relationship between BCSCs and capecitabine resistance as the consequence of capecitabine metabolism complexity.

Based on clinical observations, multidrug resistance is the main form of chemotherapy resistance. For example, paclitaxel-resistant BC often shows resistance to anthracycline at the same time (Lee et al., 2006). The main reason is that ATP-binding box transporters take part in both paclitaxel and anthracycline metabolisms, increasing the expression of drug-resistant proteins, such as MDR-1 (Genovese et al., 2017). Meanwhile, studies found that BCSCs that have DNA mismatch repair function ability, caused resistance to both anthracycline and platinum chemotherapy, but failed to resist to paclitaxel (Fedier et al., 2001). In brief, multiple pathways in BCSCs regulated the activation of metabolism and induced resistance to multiple chemotherapeutic drugs in BC, such as paclitaxel, anthracyclines, platinum, and capecitabine. Thus, it is expected that highly effective drugs targeting BCSCs emerge as a new therapeutic strategy for multi-chemotherapeutic resistance.

### Resistance to Endocrine Therapy

Endocrine therapy (ET) is a highly effective treatment for estrogen receptor (ER) positive BC by blocking ER pathway and depriving the tumor of estrogen (Howell, 2008). As a matter of fact, the ER signaling pathway is a complicated biological pathway that regulates many functions, such as cell proliferation, invasion, and angiogenesis, and is used as a crucial survival pathway by BC cells (Manavathi et al., 2013). Different endocrine therapies work by various mechanisms, which can be divided into three different categories: selective estrogen receptor modulators (SERMs), aromatase inhibitors (AI), and CDK4/6 inhibitors. Currently, evidence continues to show that BCSCs are responsible for tumor evolution and play a crucial role in achieving ET resistance (Dey et al., 2019; Rodriguez et al., 2019).

#### Tamoxifen Resistance

Tamoxifen is one of the most famous selective ER modulators, which can antagonize the effects of estrogen and bind in the ER pathway to some particular target genes (Frasor et al., 2004). Thereby, adjustment of each element or transcription in ER pathway can mediate resistance to endocrine treatment by modulating ER activity or by acting as an escape pathway. Primitively, the increase of BCSCs in advanced BC indicated their potential role in tumorigenesis and tamoxifen resistance (Pece et al., 2010). Further, recent studies demonstrated that tamoxifen resistant MCF-7 (TAM-R) cells contained a higher proportion of BCSCs than non-resistant cells (Wang et al., 2012). Therefore, we speculate that BCSCs may play an important role in endocrine resistance, and accumulating studies have confirmed this.

Recent studies provide more direct evidence on BCSCs participating in tamoxifen resistance through some important pathways. The ER signaling pathway functions as a major mechanism responsible for tamoxifen resistance. The expression of ER splicing variants, such as the estrogen related receptors and the identified short variant ERα36, have also contributed to a poor tamoxifen response (Zhang and Wang, 2013). Although considered ERα negative, BCSCs can still be stimulated by estradiol via paracrine mechanisms. A study also showed that ERα could mediate the rapid estrogen signaling in BCSCs and enhance transcription of genes related to stem cells (Gelsomino et al., 2018). ER could also promote the development of BCSCs via a crosstalk with Sox2 (Zhang Y. et al., 2012). In return, Sox2 could promote the non-genomic estrogen-stimulated activity of ER, thus inducing ER phosphorylation at Ser118 site (Zhang Y. et al., 2012; Vazquez-Martin et al., 2013). In fact, phosphorylation, ubiquitination, and other post-translational modifications of ER and its co-regulators affect the sensitivity to different endocrine therapies (Musgrove and Sutherland, 2009). However, the role of estrogen receptors β (ERβ) in BCSCs is still partly unclear, requiring further experiments to explore its relationship with endocrine resistance and BCSCs.

Another important category of pathways involved in endocrine resistance is the growth factor family. Up-regulation of EGFR, HER2, FGFR, and IGF1 receptors (IGF1R) could activate the downstream signaling pathway, especially PI3K pathways, causing tamoxifen resistance (Chakraborty et al., 2010; Arpino et al., 2008). Lately, using gene expression analysis, it was revealed that the activation of the PI3K/AKT/mTOR pathway and the inactivation of the PTEN tumor suppressor were the major alterations in MCF7 cell-derived BCSCs-enriched cells, compared to non-enriched cells. Down-regulation of PI3K, AKT1 and PI3K/mTOR reduced the self-renewal and survival of BCSCs *in vitro* and their tumor initiation and self-renewal ability *in vivo* (Gargini et al., 2015). In general, these data suggest that some regulators, such as IGF1R and PI3K, may be potential targets to recover the resistance to tamoxifen by restraining BCSCs survival and activity.

Alterations in genes involved in stemness-related pathways, such as Wnt/β-catenin, Notch, and Sonic Hedgehog, have been proven highly effective in acquiring tamoxifen resistance. According to recent studies, activation of Wnt and Notch signaling pathways induced tamoxifen resistance and promoted BCSCs activity in MCF-7 (TAM-R) cells, while inhibition of these pathways abolished the resistance (Magnifico et al., 2009; Loh et al., 2013; Lombardo et al., 2014), supporting the important role of BCSCs in endocrine-independent and TAM-resistant proliferation. Furthermore, clinical data demonstrated that upregulation of the HH signaling was related with a reduction in overall survival and recurrence-free survival in estrogen receptor positive BC patients, even leading to tamoxife resistance (Ramaswamy et al., 2012). By contrast, the stem cell-like population, cell migration, and invasion declined greatly by the inhibition of the HH signaling, thus preventing the progress of tamoxifen resistance (Ramaswamy et al., 2012). Collectively, accumulating evidence reveals complicated mechanisms with overlapping networks of tamoxifen resistance, which partly results from BCSCs-induced evolution, regulated by Notch, Wnt/β-catenin, HH, and other crucial signaling pathways.

#### Fulvestrant Resistance

Fulvestrant, a new kind of ER downregulator, can effectively reduce the level of ER in BC cells (Dowsett et al., 2005). Actually, fulvestrant was identified as an effective antagonist to endocrine-sensitive BC after failure of previous tamoxifen or aromatase inhibitor therapies (Howell and Robertson, 1995). Although the detailed mechanisms of fulvestrant resistance remain unclear, some pathways, including EGFR/ErbB2, MEK/ERK, NF-kB, PI3K-AKT, and β-catenin, have been associated with development of fulvestrant resistance (McClelland et al., 2001; Gu et al., 2002; Fan et al., 2006). It is interesting that these proteins and pathways are also correlated with the induction and maintenance of BCSCs (Hardt et al., 2012; Harrison et al., 2013; Luo et al., 2015; Majumder et al., 2016). Therefore, we speculate that BCSCs may mediate fulvestrant resistance through these pathways, but further evidence is needed to prove this.

Studies showed that resistance was associated with G protein-coupled estrogen receptor-1 (GPER) and CDK6 overexpression (Giessrigl et al., 2013; Alves et al., 2016). GPER, mediating estrogen-induced proliferation breast epithelial cells, is also essential for the survival of BCSCs (Chan et al., 2020). Recently, a study showed that microRNA-221 contributed to fulvestrant resistance via activation of β-catenin in BC and promoted the generation of BCSCs, stimulating the production of an invasive phenotype that predicts adverse outcomes (Roscigno et al., 2016). Unfortunately, few studies on fulvestrant resistance have been reported; however, the relationship between fulvestrant resistance and BCSCs may become clearer with future research.

#### Aromatase Inhibitors Resistance

Aromatase inhibitors (AIs) can inactivate aromatase, block aromatase reaction, inhibit estrogen production, and reduce estrogen levels in the blood, being an ideal ET drugs for ER+ BC in postmenopausal women. Three AIs, such as exemestane, letrozole, and anastrozole, exhibited similar resistance mechanisms in ET (Francis et al., 2015). Besides, AIs could modulate the action of androgen through the androgen receptor (AR) as well, thereby inhibiting estrogen-dependent BC growth (Macedo et al., 2006; Takagi et al., 2010). The application of AIs greatly reduced the risk of BC recurrence among postmenopausal women (Magnani et al., 2013). However, AIs resistance inevitably reduces clinical benefits. Multiple mechanisms contribute to AI resistance, involving either estrogen-independent ER growth or ER-independent activation. Among these, the PI3K pathway is a significant therapeutic target. A previous study revealed that these BCSCs showed low ER expression and the activation of PI3K signaling pathway (Hardt et al., 2012), both of which eventually led to AIs resistance (Marsden et al., 2009). Actually, the alpha-specific PI3K inhibitors, such as buparlisib, alpelisib, and taselisib, were currently utilized as novel drugs for AIs-resistant BC in phase III clinical trials (NCT02437318, NTC01610284, NCT02340221).

Stromal cells, extra-cellular matrix (ECM), and other micro-environment conditions (such as hypoxia and acidity) are also responsible for the generation of BCSCs phenotypes and endocrine (AI and TAM) resistance (Generali et al., 2006; Semenza, 2015). A lot of soluble factors that promote tumor growth and vascularization, such as transforming growth factor-β (TGFβ), which induces epithelial-to-mesenchymal transition (EMT), are secreted by cancer-associated fibroblasts (CAFs). Furthermore, downstream signaling pathways, especially PI3K and MAPK pathways, are activated by EGFR and CXCR4, thus inducing endocrine resistance (Loh et al., 2013; Ma et al., 2015). Additionally, CXCR4 was found to enhance BCSCs self-renewal by the activation of PI3K/AKT and MAPK pathways and promoted tumorigenesis through hydrocarbon receptor (AhR) signaling (Dubrovska et al., 2012). Mesenchymal stem cells (MSCs) protected cancer cells from hormone treatment through direct cell interaction and by secreted proteins (Rhodes et al., 2010). In conclusion, the tumor microenvironment is frequently linked to endocrine resistance, partly due to self-renew and maintenance of BCSCs.

### Resistance to Targeted Therapy

HER2 is a receptor tyrosine kinase which is over-expressed or genetically amplified in 15–25% of invasive BCs. As we have seen, anti-HER2 drugs, such as trastuzumab and lapatinib, have obviously improved clinical outcomes in HER2-positive BC patients. Yet the emergence of resistance to anti-HER2 drugs becomes a main barrier during the treatment of HER2-positive BC. In order to improve the prognosis of HER2-positive BC patients, it is essential to study the mechanisms of resistance to anti-HER2 therapy (Chihara et al., 2017). Several observations suggested that the resistance to anti-HER2 drugs may be driven by CSCs (BCSCs) (Martin-Castillo et al., 2013; Seo et al., 2016). Therefore, we would like to find out how BCSCs participate in resistance to anti-HER2 drugs in HER2-positive BC.

#### Trastuzumab Resistance

Trastuzumab is a molecular targeting drug for HER2 tyrosine kinase receptor. The application of trastuzumab has dramatic therapeutic efficacy in HER2+ BC, but the emergence of drug resistance hinders its clinical benefits. Multiple evidence shows that the mutation of PI3KCA (Berns et al., 2007; Dave et al., 2011) and loss of PTEN (Nagata et al., 2004; Koninki et al., 2010; Gallardo et al., 2012) leads to trastuzumab resistance in BC. Indeed, trastuzumab resistance was also associated with CSCs. CSCs may induce drug resistance via the activation of PI3K/AKT, JAK/STAT3 and NF-kB pathways (Wang et al., 2017). Meanwhile, PTEN loss and PI3KCA mutation could lead to abnormal activation of the downstream PI3K/Akt/mTOR pathway, which in turn, regulates BCSCs pool (Dey et al., 2019). Similarly, PTEN down-regulation increased BCSCs population through Akt activation of Wnt signaling pathway (Korkaya et al., 2012). We can speculate that the loss of PTEN and the mutation of PI3KCA lead to the activation of downstream PI3K/Akt/mTOR pathway in BCSCs, which results in trastuzumab resistance. Another mechanism of trastuzumab resistance was the activation of IL-6 inflammatory loop mediated BCSCs expansion, resulting in drug resistance of BC to trastuzumab. Meanwhile, IL-6 was found to inhibit PTEN when activating Akt, STAT3, and NF-kB pathways (Korkaya et al., 2012). Interestingly, STAT3 activation led to an increase in stem cell properties, which caused over-expression of HER2 and trastuzumab resistance (Chung et al., 2014). Thus, targeting upstream of JAK/STAT3 pathway, for instance IL-6 receptor antibody, could inhibit trastuzumab resistance and reduce the CSC population. A previous study showed that an excellent functional biomarker for trastuzumab resistance is Mucin1 (MUC1), and its cleaved form is named MUC1* (Sand et al., 2020). Interestingly, anti-MUC1* was found to have a dramatic, stimulatory effect on stem cell growth (Hikita et al., 2008). Fessler demonstrated a significant increase in the number of MUC1* in trastuzumb resistant cell lines (Fessler et al., 2009). In conclusion, MUC1* may be a target for reversing drug resistance of trastuzumab. Among these mechanisms, it is not difficult to find that CSCs are critical in trastuzumab resistance. Thus, the BCSCs-targeted strategy may be worth further research in recovering sensitivity of trastuzumab in BC, and may bring benefits to patients at risk of BC recurrence.

#### Lapatinib Resistance

Lapatinib is an oral small molecule drug, which targets both epidermal growth factor receptor (EGFR) and human epidermal growth factor receptor 2 (HER2). Its resistance involves many factors, such as the pathways of receptor tyrosine kinase, non-receptor tyrosine kinase, CSCs, microRNA, tumor metabolism, among others (Shi et al., 2016). MiR-205-5p is a highly conserved miRNA involved in cell differentiation, migration, and proliferation, which was found to be highly expressed in BCSCs. Moreover, it leads to lapatinib resistance by directly repressing HER2 and indirectly inhibiting EGFR (De Cola et al., 2015; Xiao et al., 2019). It was speculated that the lapatinib resistance caused by miR-205 was via the activation of PI3K/AKT signaling pathway. Therefore, down-regulating the expression of miR-205-5p contributed to inhibit the lapatinib resistance in BCSCs. The other resistance mechanism for lapatinib was associated with CD44+/CD24−, which are surface markers of CSCs (Dey et al., 2019). Knocking down CD24 could not only increase the sensitivity of HER2-positive BC cells to lapatinib, but also inhibit Akt phosphorylation (Hosonaga et al., 2014). For this reason, CD24 may be a target to reverse lapatinib resistance in BC. Actually, the use of lapatinib greatly improves BC prognosis. Nevertheless, clinical evidence suggested that lapatinib resistance led to poor therapeutic efficacy in HER2-positive BC patients. As described in the above mechanisms, CSCs seem to be the key to solve lapatinib resistance. Consequently, further understanding of the regulatory mechanisms of CSCs in lapatinib resistance in BC is essential for developing targeting strategies.

Here, we summarize the resistance mechanism of anti-HER2 drugs. The review suggested that the resistance of anti-HER2 drugs usually occurred by inducing CSC characteristics. TGFβ is a transforming growth cytokine and SMAD is an effector transforming factor in TGFβ signaling pathway. The acquisition of malignant features, such as EMT, cancer cell stemness, and drug resistance in cancer cells was closely related to TGFβ-SMAD3 signaling pathway. Sustained stimulation of TGFβ could induce SMAD3 to phosphorylate intensely and enhance the CSC traits of BC, thereby leading to HER2-positive BC resistance. Therefore, TGFβ-SMAD3 pathway plays a vital role in inducing and maintaining resistance to anti-HER2 drugs (Chihara et al., 2017). BCSCs undoubtedly participate in the process of resistance to HER2-positive BC too. Targeting BCSCs may be a possible way for us to solve the problem of resistance to anti-HER2 drugs.

### Therapeutic Strategies for Targeting Breast Cancer Stem Cells to Reverse Resistance

**FIGURE 4:**
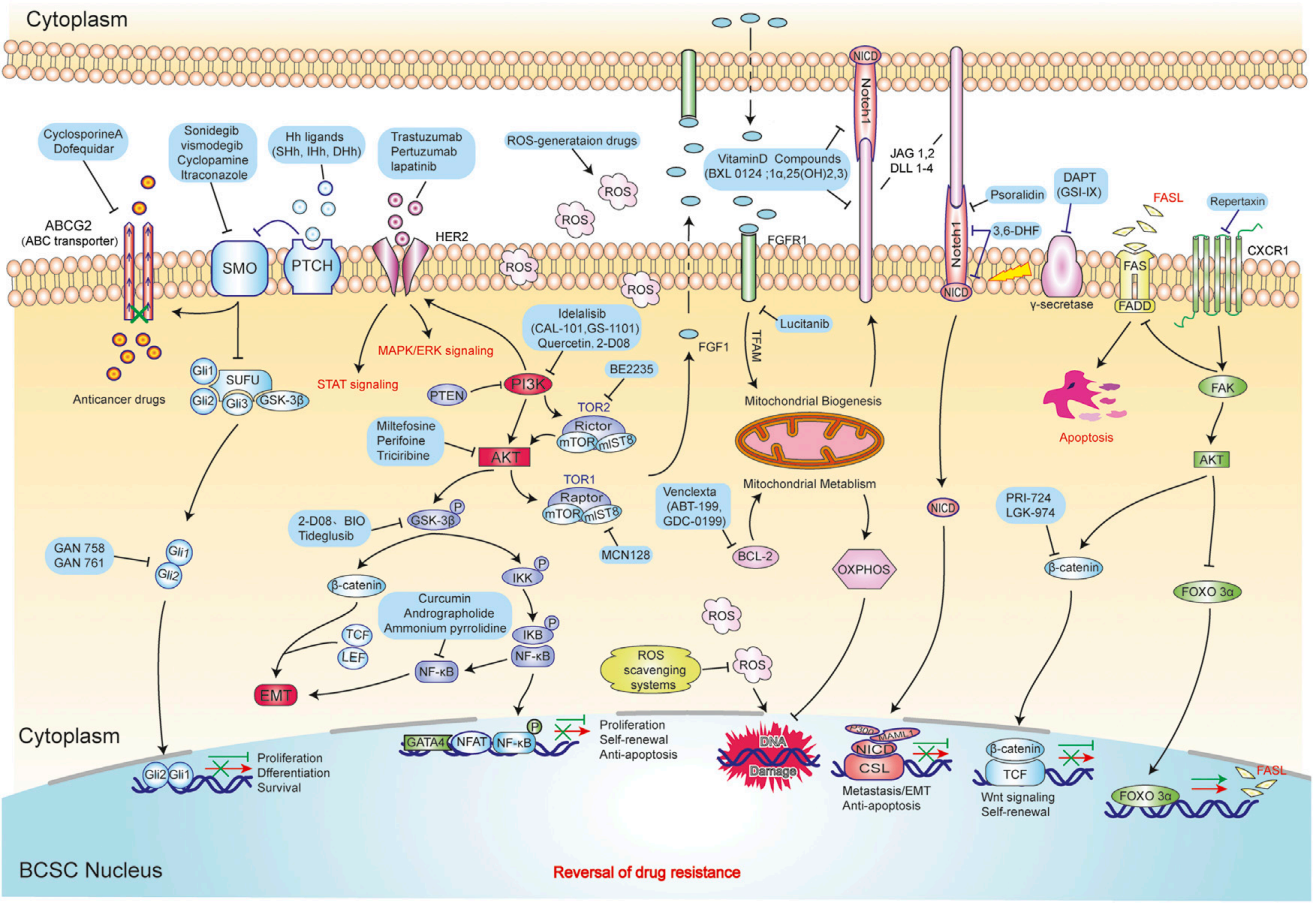
The novel strategies of drug resistance reversal in BCSCs. These strategies mainly include inhibited proliferation ability (Hh, NF-κB signaling pathway) and self-renewal ability (EMT and Wnt/β-catenin signaling pathway), promoted DNA damage (ROS scavenging system and Mitochondrial metabolism) and apoptosis (Notch and FOXO/FASL/FAS signaling pathway).

Drug resistance has turned out to be one of major problems in BC therapy, while recent studies found that BCSCs are shown to be the culprit for this phenomenon. Nevertheless, the mechanisms of drug resistance mediated by BCSCs have not been fully understood. Currently, the following vital mechanisms are recognized to be related to treatment resistance, which include overexpression of ATP-binding cassette (ABC) transporter and ALDH1, enhanced DNA repair mechanism, an altered cell cycle, resistance to apoptosis, and all microenvironment influences (Rebucci and Michiels, 2013; Smalley et al., 2013; Cojoc et al., 2015). Therefore, targeting these mechanisms may help us develop new therapies for BCSCs to reverse drug resistance in BC. We will discuss some of the current ways used to target BCSC below. The novel of therapeutic strategies for reversing drug-resistance in BCSCs are displayed in Table 3 and Figure 4.

**TABLE 3:** Therapeutic strategies to reversing drug-resistance in BCSCs.

Drug/Compound	Target	Mode of action	*In vitro* or *in vivo* or clinical trial	References
Surface markers				
HA-decorated nanoparticles and salinomycin	CD44	Increases efficiency of drug delivery by the system of CD44-HA-Nanoparticles loaded with salinomycin	*In vitro*	(Muntimadugu et al., 2016)
Doxycycline	CD44, ALDH1	Inhibits BCSCs by apoptosis	Clinical trial	(Scatena et al., 2018)
Lentivirus-mediated CD44 shRNA	CD44	Sensitizes BCSCs to doxorubicin	*In vitro*	(Hu et al., 2016)
CD133-targeted polymeric nanoparticles	CD133	Reduces tumor initiating cell by conjugating anti-CD133 monoclonal antibody to nanoparticles	*In vivo*	(Swaminathan et al., 2013)
scFv- PE38KDEL	CD133	Promotes BCSCs apoptosis by inducing cytotoxicity	*In vitro* and *in vivo*	(Ohlfest et al., 2013)
Quercetin	ALDH	Inhibits expression of Sox2, Oct4, nanog, and Bmi-1	*In vitro*	Wang R. et al. (2018)
Withaferin A			*In vitro*	Kim and Singh (2014)
Benztropine mesylate		Inhibits sphere formation and self-renewal of BCSCs	*In vitro* and *in vivo*	(Cui et al., 2017)
Deptropine citrate				
Signaling pathway				
Cyclopamine	Hedgehog signaling	Suppresses the activation of the SMO transmembrane receptor protein	*In vivo*	(Kubo et al., 2004)
Monoclonal antibody (5E1)		Inhibits breast cancer growth and metastasis.	*In vivo*	(O’Toole et al., 2011)
Nitidine chloride		Inhibits the stemness of BCSCs by downregulates the marker of CD44	*In vitro*	(Sun et al., 2016)
GANT61 (gli protein inhibitor)		Inhibits expression of glioma-associated oncogene in the Hh signaling pathway	*In vitro*	(Koike et al., 2017)
Vismodegib		Sensitizes BC cells to commonly used chemotherapy drugs by the inactivation of Hedgehog signaling	*In vivo*	(Hui et al., 2013; Palomeras et al., 2018)
Sonidegib		Inhibits the expression of BCSCs markers to sensitize BC cells to docetaxel	*In vitro* and *in vivo*	(Cazet et al., 2018; Palomeras et al., 2018)
NVPBGT226	PI3K signaling	Novel ATP-competitive mTOR kinase inhibitors for advanced breast cancer	*In vitro* and *in vivo*	(Cidado and Park, 2012)
Perifosine		Restores Tamoxifen sensitivity in resistant breast cancer cells	*In vitro*	(Farahmand et al., 2018)
Everolimus (RAD001)		Sensitizes advanced breast cancer to aromatase inhibitor	Clinical trial	(Baselga et al., 2012)
MK2206		Inhibits growth and induces breast cancer cells apoptosis	Clinical trial	(Chien et al., 2019)
PF-03084014 (nirogacestat)	Notch signaling	Sensitizes BCSCs to known chemotherapy drugs by blocking notch signaling	*In vitro* and clinical trial	Zhang C. C. et al. (2012); (Zhang and Grivennikov 2013); Locatelli et al., (2017); Ocana et al., 2010)
MK-0752		Promotes the sensitivity of BCSCs to docetaxel by strong modulation of Notch signaling	*In vitro*, *in vivo* and clinical trial	(Aktas et al., 2009; Schott et al., 2013; Venkatesh et al., 2018)
LY3039478 (crenigacestat)		γ-secretase inhibitor to promote inactivation of notch signaling	Clinical trial	(McCartney et al., 2018)
Capsaicin		Inhibits the entry of NICD to nuclear	*In vitro*	(Shim and Song, 2015)
Psoralidin		Promotes apoptosis and inhibits BCSCs proliferation and repairing	*In vitro*	(Suman et al., 2013)
RO4929097 (RG-4733)		γ-secretase inhibitor to promote inactivation of notch signaling	Clinical trial	(Strosberg et al., 2012; Koury et al., 2017; Venkatesh et al., 2018)
Foxy-5	Wnt/β-catenin	Simulates the effect of Wnt5a to inhibits metastasis	*In vivo*	(Canesin et al., 2017; Palomeras et al., 2018; Goldsberry et al., 2019)
Sulforaphane		Inhibits BCSCs self-renewal by the downregulation of the wnt/β-catenin signaling.	*In vitro*	(Li et al., 2010)
Microenvironment				
AMD3100 (CXCR4 antagonist)	SDF-I/CXCR4	Inhibits BCSC self-renewal and maintenance	*In vitro*	Liu B. Q. et al. (2017)
Reparixin	CXCR signaling	Induces BCSCs apoptosis through FASL/FAS signaling	*In vitro* and clinical trial	(Schott et al., 2013)
Evofosfamide (TH-302)	Hypoxia	Suppresses BC growth by selectively cytotoxic	*In vitro* and *in vivo*	(Liapis et al., 2016)
Echinomycin	Hypoxia response element	Reduces cytotoxic in breast cancer cells	*In vitro*	(Lathan and Von Hoff, 1984)
Tumor metabolism				
VLX600	Mitochondrial OXPHOS	Makes BCSCs death by inhibiting BCL-2	*In vitro*	(Dey et al., 2019)
Etomoxir	Carnitine palmitoyltransferase-1 inhibitor	Activates metabolic by cAMP-induced	*In vitro*	(Manerba et al., 2019)
Salinomycin	Sodium potassium gradient	Selectively eradicates BCSCs selectively via lysosomal iron Targeting.	*In vitro*	(Versini et al., 2020)
XCT790	ERRn-PGC1	Targets FOXM1 and mitochondrial biogenesis to block both the survival and propagation of BCSCs	*In vitro*	(De Luca et al., 2015)
Others				
MS-209	P-glycoprotein	Makes BCSCs more sensitive to docetaxel	*In vitro* and *in vivo*	(Naito et al., 2002)
Glucosamine	STAT 3	Inhibits BCSCs the ability to form mammosphere	*In vitro*	(Hosea et al., 2018)
Apigenin	Hippo	Inhibits BCSCs migration and metastasis by downregulating transcription activity of TAZ and YAP1	*In vitro* and *in vivo*	(Li et al., 2018)
MLN4924	Sox-2	Suppresses stem cell property and makes breast cancer cells more sensitive of tamoxifen	*In vitro*	(Yin et al., 2019)
MRX34	MiR-34a	Contains miR-34a mimic and a lipid vector and inhibits cellular proliferation, invasion and tumor sphere formation.	*In vitro*, *in vivo* and clinical trial	(Adams et al., 2016; Mohammady et al., 2019)
αEPCR-1535	Protein C receptor	Attenuates tumor growth	*In vitro*	(Schaffner et al., 2013)

NICD, Notch intracellular membrane domain.

#### Targeting Signaling Pathways

Hedgehog (Hh) signaling is a crucial regulator of proliferation, maintenance, and self-renewal of BCSCs. There is a link between the activation of HH signaling and the over expression of MDR1 and ABCG2 in BCSCs. Targeting ABCG2 or MDR1 with cyclosporin A, through inhibition of HH signaling, has shown to regulate and decrease the expression of ABCG2 and ABCG5 (Mao and Unadkat, 2015; Sims-Mourtada et al., 2015). HH signaling showed aberrant activation in Tamoxifen resistant cell lines; instead, knocking down the HH pathway can inhibit growth of tamoxifen resistant cells (Bhateja et al., 2019). Currently, two smoothened (SMO) inhibitors have made their way to clinical trials: GDC-0449 (vismodegib) with paclitaxel, epirubicin, and cyclophosphamide (NCT02694224), and LDE225 (sonidegib) combined with Docetaxel (NCT02027376). Both of the drugs were tested in triple negative BC (Hui et al., 2013; Cazet et al., 2018). It seems that oral HH inhibitors appear to be fairly safe throughout clinical testing.

Confirmatory evidence has recently revealed that the PI3K/Akt/mTOR pathway plays a significant role in regulating BCSC pool. A study observed that Akt signaling altered the subcellular localization of BCRP, thereby regulating drug efflux activity in CSCs. Inhibitors of PI3K, which can not only be blocked via Akt signaling, resulted in the suppression of cancer cell proliferation, but also enhanced the sensitivity of chemoresistant cells (Hu et al., 2008). Another observation suggested that suppressing Akt that is downstream of HER2 signaling might efficiently target BCSCs in HER2-resistant tumors (Korkaya et al., 2009). Consequently, a series of PI3K and Akt selective inhibitors, which are being clinically investigated, demonstrates promising prospects.

Notch signaling is another pathway associated with treatment resistance. miR-34a regulates Notch-1 pathway in sustaining stem cell properties of BCSC populations, thereby suggesting that the miR-34a/Notch-1 pathway might be a potential therapeutic target for treating BC (Chen et al., 2016). Activation of Notch signaling is regulated by a proteolytic enzyme (γ-secretase), so γ-secretase inhibitor is the most clinically promising candidate in reversing drug resistance (Real and Ferrando, 2009). Psoralidin had been shown to effectively inhibits BCSCs proliferation and self-renewal through downregulating Notch1 signaling (Suman et al., 2013). Besides, vitamin D compounds showed activity against BCSCs by impeding the expression of Notch signaling components, such as Notch1, Notch2, Notch3, JAG1, and JAG2 (Shan et al., 2017). Meanwhile, a study showed that targeting FGFR mitochondrial metabolism-Notch1 axis may be effective to abrogate drug-resistant CSCs in TNBC (Bhola et al., 2016). Hence, Notch signaling pathway plays an important role in drug resistance mediated by BCSCs.

#### Targeting Tumor Microenvironment

G-protein coupled receptors (GPCRs) are very important in the survival of BCSCs before and after the chemotherapy process. Chemokine receptors CXCR1 and CXCR2 generally play a role in chemotaxis of neutrophils, macrophages, and endothelial cells in a physiological microenvironment. Antagonizing CXCR1 by CXCR1-neutralizing antibody or by the small molecule inhibitor repertaxin selectively depleted more BCSCs than bulk tumor cells *in vitro*. This was followed by massive apoptosis of bulk tumor cells through FASL/FAS signaling via FAK/AKT/FOXO3A pathway (Ginestier et al., 2010). Repertaxin has already shown satisfactory effects in Phase I trials. Moreover, the chemokine receptor CXCR4 is expressed in BCSCs and forms a target in restraining or removal of BCSCs. Activation of this receptor is thought to facilitate the metastasis of mesenchymal BCSCs. CXCR4 probably stimulated the extracellular signal regulated kinase (ERK) pathway in BCSCs by activating PKA/MAPKAP2 pathway (Yi et al., 2014), thus providing resources for the research of BCSC-targeted cancer therapy through blocking these pathways by inhibiting receptors.

#### Targeting Breast Cancer Stem Cell Metabolism

The induction of oxidative stress is an important mechanism of action for many anticancer agents. BCSCs possess a highly active DNA repair system, which repairs DNA damages, particularly after chemotherapy treatment. Previous trials suggested that the ability of BCSCs to repair DNA damage is significantly related to reactive oxygen species (ROS), the levels of ROS are markedly lower in BCSCs than in non-CSCs (NCSCs) due to the high expression of free radical scavenging systems in BCSCs, such as superoxide dismutase, catalase, and glutathione peroxidase, which keep them from genotoxic damage of ROS. Thus, reduction of ROS scavengers in BCSCs markedly decreased their clonogenicity and resulted in therapeutic sensitization (Phillips et al., 2006; Diehn et al., 2009). Through H_2_O_2_-induced BCSC loss of function, ROS-generating drugs may have the therapeutic potential to eradicate drug-resistant BCSCs via induction of premature senescence (Zhong et al., 2019). Moreover, increasing mitochondrial activity is associated with resistance to DNA damage in BC. BCSCs are obviously dependent on glucose and mitochondrial metabolism. BCL-2 protein is a famous regulator of mitochondrial metabolism, inhibition of BCL-2 can result in the inhibition of oxidative phosphorylation (OXPHOS), which will lead to the reduction of BCSCs depending on OXPHOS (Deshmukh et al., 2016).

Besides potentiated ROS scavenging systems, BCSCs can protect themselves from several chemotherapeutic drugs which target the cell cycle process by maintaining a quiescent state in G0 phase (Yoshida and Saya, 2016). BCSCs can adopt dormancy-associated phenotypes through upregulating autophagic pathways (Vera-Ramirez et al., 2018). Salinomycin is a kind of ionophore antibiotic, which has been shown to be effective in clearing BCSCs through autophagy (Jiang et al., 2018). Recently, studies showed that the mechanistic link between autophagy and metastastic dormancy was associated with Spleen Tyrosine Kinase (SYK) in epithelial-mesenchymal transition (EMT) required for BC metastasis. Fostamatinib, a SYK pharmacologic inhibitior, prevents mesenchymal-epithelial transition (MET), which can inhibit metastatic tumor outgrowth (Shinde et al., 2019). Currently, tyrosine kinase inhibitors are being tested in clinical trials.

#### Nano-therapeutics Against Breast Cancer Stem Cell

Nanoparticle (NP)-mediated therapy is an effective delivery strategy for cancer therapeutics. It contributes to specific delivery of a chemotherapeutic drug, RNAi, or antibodies to the stem cell population by recognizing antibodies/aptamers against BCSC-specific markers.

CD44 is the first discovered and the most commonly used surface marker of BCSCs, which plays an important role in all aspects of tumor cells, such as growth and proliferation, migration, differentiation, apoptosis, self-renewal, microenvironment, EMT, and drug resistance (Jin et al., 2017). As a cell receptor, CD44 mediates the communication with the microenvironment through interacting with certain extracellular ligands. For the past few years, the development of an antibody against CD44, which could induce BCSCs terminal differentiation, had already been found to be effective and has been gradually accepted (Naor et al., 1997). In aggressive BC, the combination of anti-human CD44 monoclonal antibody with doxorubicin and cyclophosphamide using NPs has been used to prevent tumor recurrence (Fan et al., 2010; Wu et al., 2017).

Micro RNAs (miRs) play a key role in the sustenance and heterogeneity of BCSCs in BC. They can regulate proteins associated with drug resistance in human BC. For instance, miR-21 may facilitate the inhibition of tumor proliferation, growth, and migration (Han et al., 2012); miR-100 inhibits self-renewal of BCSCs and tumorigenesis (Deng et al., 2014); miR-199a can increase stem cell properties in BCSCs (Celia-Terrassa et al., 2017). miR-205-5p is highly expressed in BCSCs and is related to therapy resistance (De Cola et al., 2015). Moreover, research shows that the high expression of STAT3 affects doxorubicin resistance of BCSCs, and miR-124 reverses this resistance of BCSCs through targeting STAT3 to control the HIF-1 signaling pathway (Liu C. et al., 2019). Consequently, targeting miRs and delivering siRNAs to tumors using NPs is an effective strategy to reverse drug resistance and enhance drug efficacy.

Aldehyde dehydrogenase 1 (ALDH1) is a NAD(P)+-dependent enzyme, which is the key enzyme to oxidize intracellular aldehydes to carboxylic acids. ALDH1 is found to be highly active in BCSCs, increasing their proficiency by removing toxic oxygen radicals from the tumor microenvironment (Charafe-Jauffret et al., 2013). By consulting relevant literatures, we also found that the increased levels of ALDH family members were correlated with chemoresistance (Croker et al., 2009; Tanei et al., 2009). ALDHs inhibition sensitizes BCSCs to chemotherapy (Croker and Allan, 2012). NPs containing doxorubicin and chloroquine have been shown to reduce ALDH high population of MDA-MB-231 cells (Li et al., 2015), and several ALDH inhibitors are currently in the preclinical stage.

#### Other Therapeutic Approaches

CSCs manifest a high number of proteins on their cell surface, such as ABC transporters, ABCB1 (P-gp, MDR1), ABCG2 (BCRP1), ABCC11 (MRP8), and ABCB, which are strongly expressed in CSC’s chemo-resistance (Dean, 2009). How do CSCs develop drug resistance through the protein molecule above? In BC, a recent study has indicated that the prominently activated ATP binding cassette (ABC) or drug efflux pump of BCSCs can successfully pump out chemotherapeutic drugs, such as anthracycline or taxanes, which are known as the most essential drugs of BC treatment (Cojoc et al., 2015). Furthermore, other scholars have found that an increased level of ABCG2 in BCSCs enabled rapid expulsion of cytotoxic drugs, conferring cellular resistance to antitumor drugs (Hirschmann-Jax et al., 2004). A recent study has confirmed that SOX2-ABCG2-TWIST1 axis can promote stemness and chemoresistance in TNBC, further indicating that ABC proteins are potential targets for BCSCs eradication (Mukherjee et al., 2017). Dofequidar, an ABC transporter inhibitor, could increase the sensitivity of BCSC to anticancer drugs; it showed promising results in patients with advanced or recurrent BC when combined with other chemotherapeutic agents, such as cyclophosphamide, doxorubicin, and fluorouracil (Saeki et al., 2007). Additionally, SOX2 is a key transcription factor that plays critical roles in maintaining stem cell properties and conferring drug resistance. MLN4924 can repress the expression of SOX2, leading to suppression of stem cell properties and sensitization of BC cells to tamoxifen (Yin et al., 2019).

## Conclusion

BC remains the most frequent cancer in women, and significant public health issue globally (Zavala et al., 2019). Both of the developing and developed world are suffering from BC incidence and mortality (Global Burden of Disease Cancer Collaboration et al., 2015). Due to limitations of therapeutic strategies, it is urgent to explore novel and effective strategies. The important role of BCSCs in drug resistance, recurrence, and metastasis of BC has attracted more and more attention. Many studies have also enlightened the drug resistance mechanism of BCSCs. Currently, various treatments targeting BCSCs have been in preclinical and clinical trials. Unfortunately, the mechanism of drug resistance that is controlled by BCSC rarely functions individually. In the process of antagonizing anticancer drugs, these mechanisms interact with each other and form a complex functional network of drug resistance. Hence, inhibiting a drug resistant pathway is likely to trigger feedback mechanisms that ultimately allow BCSCs to escape the effects of the drug. Therefore, the therapy based on the combination of multiple targets for BCSCs’ functional network is the most promising approach. Furthermore, existing nanobiology technologies should be fully utilized, through finding specific surface markers of targeting BCSCs, to locate and eliminate BCSCs accurately. Recently, biologically and chemically synthesized gold nanoparticles (AuNPs) (Virmani et al., 2019), silver nanoparticles (AgNPs) (Muthupandian et al., 2019) and selenium nanoparticles (SeNPs) (Vahidi et al., 2020) have attracted significant attention for their anticancer effects against cancers such as lung cancer, colorectal Cancer (Barabadi et al., 2020a), cervical cancer (Barabadi et al., 2020b) and prostate cancer (Barabadi et al., 2019a). Fortunately, AuNPs (Barabadi et al., 2019b) and AgNPs (Saravanan et al., 2020) have also been reported to play an important role in the treatment of BC. With the development of cancer nanomedicine, it is expected that biologically and chemically synthesized NPs may emerge as potential BCSCs therapeutic agents alone or in combination with anti-cancer drugs before long of future. In conclusion, these therapies targeting BCSCs will lay the foundation for reversing drug resistance and attaining favorable prognosis in BC.

## Author Contributions

QZ and MZ conceived and drafted the manuscript. LZ and XM discussed the concepts of the manuscript. QZ drew the figures. LZ and XM approved the version to be submitted.

## Funding

This work was supported by a special program from the Ministry of Science and Technology of China (2016YFA0502500 to LZ), the Chinese National Natural Science Funds (91753139 to LZ and 81973861 to XM), the Zhejiang Natural Science Fund (LD19C070001 to LZ).

## Conflict of Interest

The authors declare that the research was conducted in the absence of any commercial or financial relationships that could be construed as a potential conflict of interest.
